# Resistance to tetracycline and β-lactams and distribution of resistance markers in enteric microorganisms and pseudomonads isolated from the oral cavity

**DOI:** 10.1590/S1678-77572009000700004

**Published:** 2009

**Authors:** Marcelle Marie Buso RAMOS, Ellen Cristina GAETTI-JARDIM, Elerson GAETTI-JARDIM

**Affiliations:** 1 Undergraduate student, School of Dentistry of Araçatuba, São Paulo State University-UNESP.; 2 DDS, MSc Student, Department of Pathology and Clinical Propedeutics, School of Dentistry of Araçatuba, São Paulo State University-UNESP.; 3 DDS, MSc, PhD, Associate Professor, Department of Pathology and Clinical Propedeutics, School of Dentistry of Araçatuba, São Paulo State University-UNESP.

**Keywords:** Oral cavity, Enteric bacteria, PCR

## Abstract

This study evaluated the occurrence of enteric bacteria and pseudomonads resistant to tetracycline and β-lactams in the oral cavity of patients exhibiting gingivitis (n=89), periodontitis (n=79), periodontally healthy (n=50) and wearing complete dentures (n=41). Microbial identification and presence of resistance markers associated with the production of β-lactamases and tetracycline resistance were performed by using biochemical tests and PCR. Susceptibility tests were carried out in 201 isolates of enteric cocci and rods. Resistance to ampicillin, amoxicillin/clavulanic acid, imipenem, meropenem and tetracycline was detected in 57.4%, 34.6%, 2.4%, 1.9% and 36.5% of the isolates, respectively. β-lactamase production was observed in 41.2% of tested microorganisms, while the most commonly found β-lactamase genetic determinant was gene *bla*_TEM_. Tetracycline resistance was disseminated and a wide scope of *tet* genes were detected in all studied microbial genus.

## INTRODUCTION

The oral microbiota is composed of more than 500 different microbial species, most of them associated with oral health. However, sometimes the balance between the host’s immune system and microbial virulence is lost and opportunistic infections may arise. Hence, oral infectious diseases have been frequently associated with alterations in the host’ immune system, poor oral hygiene, denutrition, and alcoholism^18^.

Associations between the occurrence of opportunistic and superinfecting pathogens with patients exhibiting different periodontal status^2^ or wearing complete dentures^4^ have been established. However, the role enteric bacteria and pseudomonads play in the etiology of periodontal disease needs further studies. In edentulous patients wearing complete dentures, the presence of enteric microorganisms may be associated with development of mucositis and usually reflects poor hygiene standards^4^.

Suppression of the oral microbiota by abusive or intensive use of antibiotics may facilitate a persistent colonization of the oral cavity by these opportunistic microorganisms^18^. These microorganisms may spread to microbial populations in nosocomial infections or to the dental biofilm, acting as reservoirs for antibiotic resistance genes^7^.

Tetracyclines were among the most widely used drugs in dentistry in the 80’s. Their effects on anaerobes and *Aggregatibacter actinomycetemcomitans* made these drugs the first choice in the treatment of aggressive periodontitis and necrotizing periodontitis. β-lactams, such as ampicillin, amoxicillin, cefoxitin and others constitute the basis of antimicrobial treatment of head and neck infections. However, microbial resistance to these drugs has compromised the efficacy of this therapy and the dissemination of resistance genes among oral microorganisms needs further investigation, as the oral cavity may harbor some multiresistant microorganisms, particularly enteric rods and cocci.

Thus, the aim of this study was to evaluate the presence of antimicrobial resistance genes (tetracycline and β-lactams) in enteric microorganisms isolated from the oral cavity of patients with gingivitis, periodontitis, periodontally healthy patients and patients wearing complete dentures, determining the distribution of most common β-lactamase markers and tetracycline resistance markers.

## MATERIAL AND METHODS

### Microorganisms and microbial identification

Enteric microorganisms were isolated from 250 patients (84 males and 166 females), mean age 43.03 years, within an 10-year follow-up period (1998–2008) at the School of Dentistry of Araçatuba, São Paulo State University (UNESP), Brazil. Forty-one patients wore complete dentures, 89 exhibited gingivitis, 70 chronic periodontitis and 50 were periodontally healthy. A written consent form was signed by all patients included in this study, which was approved by the Institutional Review Board of School of Dentistry of Araçatuba (Proc.27/2000 and 34/2006).

Microbial isolation was performed as previously described^6^. The isolates were identified by Gram staining, colony morphology on agar plates, catalase assay, and biochemical identification kits (BioMérieux, Marcy le’ Etoile, France). A total of 201 enteric microorganisms and pseudomonads were submitted to susceptibility tests, as follows: *Burkholderia cenocepacia* (5 isolates), *Citrobacter freundii* (7 isolates), *Enterobacter cloacae* (18 isolates), *E. intermedius* (6 isolates), *E. sakazakii* (9 isolates), *Enterococcus* sp. (18 isolates), *E. faecalis* (31 isolates), *E. faecium* (8 isolates), *Escherichia coli* (6 isolates), *Klebsiella oxytoca* (11 isolates), *K. pneumoniae* (3 isolates), *Morganella morganii* (17 isolates), *Pantoea agglomerans* (7 isolates), *Proteus mirabilis* (5 isolates), *P. vulgaris* (7 isolates), *Providencia alcalifaciens* (6 isolates), *Pseudomonas aeruginosa* (15 isolates), *P*. *fluorescens* (4 isolates), *Serratia* sp. (9 isolates), and *S. liquefaciens* (9 isolates).

All isolates were examined for susceptibility to tetracycline, ampicillin, amoxicillin/clavulanic acid, cefoxitin, cephalothin, imipenem and meropenem by the agar dilution method. When the Clinical Laboratory and Standards Institute (CLSI) antimicrobial breakpoints were not established, the breakpoints adopted by the British Society for Antimicrobial Chemotherapy (BSAC) were followed. Mueller-Hinton agar (MHA) was used for all isolates.

In the susceptibility tests, five pure colonies of each bacterial strain were inoculated into 2 ml of sterile Mueller Hinton broth and incubated at 37°C for 12–24 h. Then, the turbidity was adjusted to match the 0.5 McFarland turbidity standard. The bacterial inocula were standardized in 10^5^ cells and transferred to Mueller-Hinton agar plates containing the antimicrobial agent and control plates (without drugs), using a Steer’s replicator (Cefar Ltda., SP, Brazil). The test and control agar plates were incubated aerobically at 37°C, for 48 h.

Antimicrobials were tested in two-fold dilution series ranging from 0.06 μg/mL to 256 μg/mL. After incubation, the organisms were classified as sensitive or resistant, according to CLSI and BSAC guidelines. *E. coli* ATCC 25922, *S. aureus* ATCC 29213, *P. aeruginosa* ATCC 27853, and *E. faecalis* ATCC 29212 were used in the assays involving facultative anaerobes.

### Detection of β-lactamases

β-Lactam-resistant isolates were also tested for β-lactamase activity by both chromogenic cephalosporin and biological method^5^. These two methods were performed because nitrocefin-based β-lactamase assays have not proven useful in detecting β-lactamase production by some microorganisms. In all tests, *S. aureus* ATCC 29213 was used as the positive control for β-lactamase production.

### Distribution of antimicrobial resistance determinants

Bacterial DNA from each β-lactamase producers placed in sterile ultra-pure water was obtained by using a QIAamp DNA Mini Kit (Qiagen, Hilden, Germany). DNA concentrations were determined with a spectrophotometer at A_260_nm (Model DU-640, Beckman Instruments, Richmond, Wash, USA).

Tetracycline-resistant isolates were screened for tetracycline resistance genes^1^^,^^16^ *tet*(A), *tet*(B), *tet*(C), *tet*(D), *tet*(E), *tet*(G), *tet*(K), *tet*(L), *tet*(M), *tet*(O), *tet*(Q), *tet*(S), and *tet*(T), while β-lactam-resistant microorganisms were screened for *bla*_TEM_, *bla*_CTX-M_ and *bla*_SHV_ genes^3^^,^^8^ using specific primer pairs. DNA amplification was performed in a DNA thermal cycler (AmpliTherm Thermal Cycler, Madison, WI, USA). The amplification conditions were 94°C (5 min) for initial denaturation, followed by 35 cycles at 94°C for 1 min, annealing temperature adequate for each primer pair for 1 min and 72°C for 1 min for extension; then 72°C for 5 min to allow final DNA extension.

## RESULTS

In relation to susceptibility to antimicrobial drugs, significant levels of resistance were observed for all β-lactams, except for imipenem and meropenem, which presented 2.4% and 1.9% of resistance, respectively. Resistance to ampicillin, and cephalothin were detected in 57.4%, and 41.7% of tested bacteria, especially *Pseudomonadaceae* and *Enterobacteriaceae*. Out of 121 bacterial isolates resistant to ampicillin or amoxicillin, 87 were β-lactamase producers of (41.2% of the isolated bacteria and 72.9% of ampicillin-resistant isolates). The production of these hydrolytic enzymes seems to be the major mechanism of resistance to β-lactams, excluding most pseudomonads, and enterococci, where β-lactamases were not detected (Table 1).

**Table 1:** Resistance to β-lactams and tetracycline in enteric bacteria and pseudomonads

TAXON (N)	Resistance prevalence N (%)							β-lactamase production
	AM	AMC	CF	CP	IM	ME	TE	
*A. bamanii* (10)	6 (60.0)	1 (10.0)	2 (20.0)	3 (30.0)	0 (0.0)	0 (0.0)	2 (20.0)	6 (60.0)
*B. cenocepacia* (5)	4 (80.0)	2 (40.0)	1(20.0)	1 (20.0)	0 (0.0)	0 (0.0)	2 (40.0)	2 (40.0)
*C. freundii* (7)	4 (57.1)	2 (28.6)	3 (42.9)	3 (42.9)	0 (0.0)	0 (0.0)	2 (28.6)	4 (57.1)
*E. cloacae* (18)	14 (77.8)	9 (50.0)	11 (61.1)	11 (61.1)	0 (0.0)	0 (0.0)	1 (5.6)	13 (72.2)
*E. intermedius* (6)	2 (33.3)	2 (33.3)	0 (0.0)	0 (0.0)	0 (0.0)	0 (0.0)	1 (16.7)	2 (33.3)
*E. sakazakii* (9)	4 (44.4)	1 (11.1)	2 (22.2)	9 (100.0)	0 (0.0)	0 (0.0)	1 (11.1)	4 (44.4)
*Enterococcus* sp. (18)	4 (22.2)	0 (0.0)	6 (33.3)	7 (38.9)	0 (0.0)	0 (0.0)	7 (38.9)	0 (0.0)
*E. faecalis* (31)	6 (19.4)	0 (0.0)	3 (9.7)	12 (38.7)	0 (0.0)	0 (0.0)	19 (61.3)	0 (0.0)
*E. faecium* (8)	4 (50.0)	0 (0.0)	4 (50.0)	4 (50.0)	2 (25.0)	1 (12.5)	3 (37.5)	0 (0.0)
*E. coli* (6)	4 (66.7)	1 (16.7)	0 (0.0)	0 (0.0)	0 (0.0)	0 (0.0)	1 (16.7)	4 (66.7)
*K. oxytoca* (11)	7 (63.6)	5 (45.5)	1 (9.1)	3 (27.3)	0 (0.0)	0 (0.0)	0 (0.0)	6 (54.5)
*K. pneumoniae* (3)	3 (100.0)	2 (66.7)	0 (0.0)	0 (0.0)	0 (0.0)	0 (0.0)	0 (0.0)	3 (100.0)
*M. morganii* (17)	12 (70.6)	9 (52.9)	5 (29.4)	9 (52.9)	1 (5.9)	1 (5.9)	7 (41.2)	9 (52.9)
*P. agglomerans* (7)	6 (85.7)	6 (85.7)	3 (42.9)	3 (42.9)	0 (0.0)	0 (0.0)	2 (28.5)	6 (85.7)
*P. mirabilis* (5)	3 (60.0)	0 (0.0)	0 (0.0)	2 (40.0)	0 (0.0)	0 (0.0)	2 (40.0)	4 (80.0)
*P. vulgaris* (7)	5 (71.4)	2 (28.6)	1 (14.3)	1 (14.3)	0 (0.0)	0 (0.0)	1 (14.3)	5 (71.4)
*P. alcalifaciens* (6)	4 (66.7)	4 (66.7)	1 (16.7)	2 (33.3)	0 (0.0)	0 (0.0)	4 (66.7)	4 (66.7)
*P. aeruginosa* (15)	13 (86.7)	13 (86.7)	8 (53.3)	9 (60.0)	2 (13.3)	2 (13.3)	11 (73.3)	3 (20.0)
*P. fluorescens* (4)	3 (75.8)	3 (75.0)	0 (0.0)	1 (25.0)	0 (0.0)	0 (0.0)	1 (25.0)	1 (25.0)
*S. liquefaciens* (9)	6 (66.7)	6 (66.7)	2 (22.2)	3 (33.3)	0 (0.0)	0 (0.0)	5 (55.6)	6 (66.7)
*Serratia* sp. (9)	7 (77.8)	5 (55.6)	5 (55.6)	5 (55.6)	0 (0.0)	0 (0.0)	5 (55.6)	6 (66.7)
Total (211)	121 (57.4)	73 (34.6)	58 (27.5)	88 (41.7)	5 (2.4)	4 (1.9)	77 (36.5)	87 (41.3)

AM= ampicillin; AMC= amoxicillin/clavulanic acid; CF= cefoxitin; CP= cephalotin; IM= imipenem; ME= meropenem; TE= tetracycline

Most of β-lactamase Gram-negative producers harbored β-lactamases. The detection of antimicrobial resistance determinants evidenced that 29.9% of Gram-negative isolates resistant to ampicillin harbored *bla*_TEM_ genes, while *bla*_SHV_ and *bla*_CTX-M_ were detected in 23.4% and 2.8% of the resistant isolates, respectively (Table 2). These genes were not detected in enterococci (Table 3).

**Table 2:** Distribution of tetracycline and β-lactam resistance genes in resistant Gram-negative isolates

Resistant microorganisms	Frequency of antimicrobial resistance determinants N(%)								
	*bla*_TEM_	*bla*_CTX-M_	*bla*_SHV_	*tet*(A)	*tet*(B)	*tet*(C)	*tet*(D)	*tet*(E)	*tet*(M)
*A. bamanii*	3 (50.0)	0 (0.0)	3 (50.0)	0 (0.0)	2 (100.0)	0 (0.0)	0 (0.0)	0 (0.0)	0 (0.0)
*B. cenocepacia*	0 (0.0)	0 (0.0)	1 (50.0)	0 (0.0)	0 (0.0)	0 (0.0)	0 (0.0)	0 (0.0)	0 (0.0)
*C. freundii*	1 (25.0)	0 (0.0)	1 (25.0)	1(50)	0 (0.0)	0 (0.0)	1 (50.0)	0 (0.0)	0 (0.0)
*E. cloacae*	5 (35.7)	1 (7.1)	3 (21.5)	0 (0.0)	1 (100.0)	0 (0.0)	0 (0.0)	0 (0.0)	0 (0.0)
*E. intermedius*	2 (100.0)	0 (0.0)	0 (0.0)	0 (0.0)	1 (100.0)	0 (0.0)	0 (0.0)	0 (0.0)	0 (0.0)
*E. sakazakii*	2 (50.0)	0 (0.0)	2 (50.0)	0 (0.0)	0 (0.0)	1 (100.0)	0 (0.0)	0 (0.0)	0 (0.0)
*E. coli*	1 (25.0)	0 (0.0)	0 (0.0)	0 (0.0)	0 (0.0)	0 (0.0)	0 (0.0)	1 (100.0)	0 (0.0)
*K. oxytoca*	1 (14.3)	1 (14.3)	1 (14.3)	0 (0.0)	2 (100.0)	0 (0.0)	0 (0.0)	0 (0.0)	0 (0.0)
*K. pneumoniae*	0 (0.0)	0 (0.0)	3 (75.0)	0 (0.0)	0 (0.0)	2 (100)	0 (0.0)	0 (0.0)	0 (0.0)
*M. morganii*	7 (58.3)	1 (8.3)	4 (33.3)	1 (14.3)	3 (42.9)	0 (0.0)	0 (0.0)	0 (0.0)	0 (0.0)
*P. agglomerans*	0 (0.0)	0 (0.0)	4 (66.7)	0 (0.0)	2 (100.0)	0 (0.0)	0 (0.0)	0 (0.0)	0 (0.0)
*P. mirabilis*	0 (0.0)	0 (0.0)	1 (25.0)	1(50.0)	0(0.0)	0 (0.0)	0 (0.0)	0 (0.0)	1 (50.0)
*P. vulgaris*	2 (40.0)	0 (0.0)	1 (20.0)	1(100.0)	0 (0.0)	0 (0.0)	0 (0.0)	0 (0.0)	0 (0.0)
*P. alcalifaciens*	2 (50.0)	0 (0.0)	0 (0.0)	0 (0.0)	2 (50.0)	0 (0.0)	0 (0.0)	1 (25.0)	1 (25.0)
*P. aeruginosa*	0 (0.0)	0 (0.0)	0 (0.0)	5 (54.5)	1 (9.09)	0 (0.0)	0 (0.0)	5 (45.45)	0 (0.0)
*P. fluorescens*	0 (0.0)	0 (0.0)	0 (0.0)	1 (50.0)	0 (0.0)	0 (0.0)	0 (0.0)	1 (50.0)	0 (0.0)
*S. liquefaciens*	2 (40.0)	0 (0.0)	1 (20.0)	3 (60.0)	0 (0.0)	0 (0.0)	2 (40.0)	0 (0.0)	0 (0.0)
*Serratia* sp*.*	4 (57.1)	0 (0.0)	1 (14.3)	3 (60.0)	0 (0.0)	0 (0.0)	2 (40.0)	0 (0.0)	0 (0.0)
Total	32 (29.9)	3 (2.8)	25 (23.4)	12 (25.0)	10 (20.8)	3 (6.3)	5 (10.4)	6 (12.5)	2 (4.2)

**Table 3:** Distribution of tetracycline, ampicillin and gentamicin resistance genes in enterococci resistant to these antimicrobials

Resistant strains	Frequency of antimicrobial resistance determinants N (%)					
	*bla*_TEM_/*bla*_CTX-M_/*bla*_SHV_					
		*tet* (K)	*tet* (L)	*tet* (M)	*tet* (O)	*tet* (S)
*Enterococcus* sp.	0 (0.0)	3 (42.9)	1 (14.3)	2 (28.6)	1 (14.3)	0 (0.0)
*E. faecalis*	0 (0.0)	6 (31.6)	2 (10.5)	7 (36.8)	3 (15.8)	1 (5.3)
*E. faecium*	0 (0.0)	3 (66.7)	0 (0.0)	1 (33.3)	0 (0.0)	0 (0.0)
Total	0 (0.0)	10 (34.5)	1 (3.5)	7 (24.1)	2 (6.9)	3 (10.3)

Resistance to tetracycline was also widely disseminated in the microbial enteric strains and 36.5% of tested microorganisms were resistant. The presence of tetracycline resistance determinants was widely disseminated among resistant Gram-negative isolates and enterococci. *Tet*(A) and *tet*(B) were the most common in Gram-negative bacteria; while *tet*(K), *tet*(M) and *tet*(O) were predominant in resistant enterococci. *Tet*(G), *tet*(Q) and *tet*(T) were not detected.

## DISCUSSION

Enteric bacteria and pseudomonads have been involved in many oral and extra-oral infections, and some studies have evidenced that the oral cavity may act as a reservoir of enteric microorganisms and their virulence genes^1^^,^^6^^,^^7^.

In spite of the small participation of enteric bacteria and pseudomonads in the total microbial load present in the gingival sulcus, supragingival biofilm, saliva and other sites of the oral cavity, the occurrence of these pathogens should not be neglected^7^. Antimicrobial resistance surveillance programs have provided sufficient data about antimicrobial susceptibility of clinically relevant enteric bacteria and pseudomonads from nosocomial infections and environment^14^^,^^17^, although few reports describe the antimicrobial susceptibility of these organisms isolated from the oral cavity^2^. In addition, information about the genetic determinants associated with this resistance is not clarified yet and most available data regards nosocomial infections, as mentioned above.

β-Lactam agents such as penicillins, cephalosporins, monobactams and carbapenems are among the most frequently prescribed antibiotics worldwide. In Gram-negative pathogens, β-lactamases remain the most important factor to β-lactam resistance, and their increasing prevalence, as well as their alarming evolution seems to be directly linked to their clinical use^14^.

In the present study, the genetic bases of β-lactamase production in enteric Gram-negative rods were mainly associated with *bla*_TEM_ gene, which evidenced a noticeable dissemination among Gram-negative enteric bacteria^10^^,^^19^. Presence of β-lactamase genetic markers was significantly more pronounced in our study than previously reported in literature, even though the distribution of particular determinants in β-lactamase-producer strains was similar^10^^,^^19^.

However, the introduction of new β-lactams with different activity spectra has led to a selection of different genes and mutations that confer resistance to these drugs, especially β-lactamase-producers, mainly in members of family *Enterobacteriaceae*. In this family, most β-lactamase producers harbor *bla*_TEM_, *bla*_SHV_ and *bla*_CTX-M_ resistance determinants^14^. Thus, the distribution of these resistance markers in enteric microorganisms distributed in the dental biofilm and mucosal surfaces remains unclear.

Therapeutic options for infections caused by Gram-negative organisms expressing β-lactamases are limited because these organisms are usually resistant to all β-lactam antibiotics, except the carbapenems. Several families of β-lactamases from Gram-negative organisms were identified, but no phenotypic test can differentiate them, impairing surveillance and epidemiological studies^13^.

The genes screened in the β-lactamase family represent only a small part of the cellular defense mechanisms that prokaryotes developed to avoid the action of β-lactams. *Enterobacteriaceae* isolates that exhibited uncertain identification by PCR were later classified as *K. oxytoca*, *Enterobacter* spp. and *C. freundii* due to detection markers of β-lactam resistance^8^. Moreover, *K. oxytoca* strains are known to express specific class A β-lactamases that were not considered in this study; while the resistance to β-lactams in *Enterobacter* sp. and *C. freundii* is generally attributed to the expression of chromosomal AmpC β-lactamases, as also described to some pseudomonads^9^. Possibly, these lactamases may be responsible for the β-lactam resistance phenotype, specifically to penicillins and narrowspectrum cephalosporins, registered in some isolates affiliated to these genera.

Enterococci in general and *E. faecium* in particular, are intrinsically more resistant to penicillin and ampicillin than the other streptococci. Ampicillin resistance in *E. faecium* is due to expression of the low-affinity class B penicillin-binding protein 5 (PBP5). Early studies suggested that higher levels of ampicillin resistance in *E. faecium* were achieved by increasing levels of PBP 5 expression. More commonly, mutations that are presumed to lower the affinity for β-lactam antibiotics have been identified within *pbp5* genes of highly resistant clinical isolates^15^. The results of the present investigation also suggested that enterococcal resistance to β-lactams, especially ampicillin, is not related to gene *bla*_TEM_, as this gene and β-lactamase activities were not detected.

Tetracycline resistance was also often observed. The most common genetic determinants of tetracycline resistance are represented by genes *tet*, which can be separated into genes that encode efflux proteins, especially genes *tet*(A), *tet*(B), *tet*(C), *tet*(D), *tet*(E), *tet*(G), *tet*(I), *tet*(K), and *tet*(L); those that protect the ribosomes from the action of tetracycline, genes *tet*(M) *tet*(O) *tet*(Q); and gene *tet*(X) that encodes a protein able to inactivate the antibiotic drug^16^. In Gram-positive cocci, the concomitant presence of two or more genes *tet* is common but this peculiarity was not confirmed in the present study, since only 5 isolates (17.2%) of enterococci expressed simultaneously *tet*(K) and *tet*(M) determinants.

In *Enterobacteriaceae*, the most common tetracycline resistance markers were *tet*(A) and *tet*(B), which were present in 45.8% of the tetracycline resistant isolates, according to previous studies^1^^,^^11^^,^^12^^,^^16^. In enterococci, genes *tet*(K) and *tet*(M) represented 58.6% of the detected resistance markers.

Heterogeneity of tetracycline resistance genes in Gram-negative enteric rods and enterococci was significant, as also previously reported^11^, although these genes were not detected in 18 enteric resistant isolates. There are several possible explanations for the non-detection of *tet* genes in 23.4% of our resistant isolates. The most probable possibility is that we screened only 12 of the 38 recognized *tet* genes and some isolates present an inherent resistance to tetracycline as opposed to acquired resistance.
